# An Autocrine Regulator Loop Involving Tumor Necrosis Factor and Chemokine (C-C motif) Ligand-2 Is Activated by Transforming Growth Factor-β in Rat Basophilic Leukemia-2H3 Mast Cells

**DOI:** 10.3390/ijms26094263

**Published:** 2025-04-30

**Authors:** Dulce Avila-Rodríguez, Alfredo Ibarra-Sánchez, Marcela Sosa-Garrocho, Genaro Vázquez-Victorio, Cassandre Caligaris, Isabel Anaya-Rubio, Deisy Segura-Villalobos, Ulrich Blank, Claudia González-Espinosa, Marina Macias-Silva

**Affiliations:** 1 Instituto de Fisiología Celular, Universidad Nacional Autónoma de México, Ciudad de México 04510, Mexico; dulceavilar@outlook.com (D.A.-R.); msosa@ifc.unam.mx (M.S.-G.); casscaligaris21@gmail.com (C.C.); ianaya@ifc.unam.mx (I.A.-R.); 2 Departamento de Farmacobiología del Centro de Investigación y de Estudios Avanzados (Cinvestav, sede Sur), y Centro de Investigación sobre Envejecimiento, Ciudad de México 14400, Mexico; aibarra@cinvestav.mx (A.I.-S.); deisy_mia@hotmail.com (D.S.-V.); 3 Facultad de Ciencias, Universidad Nacional Autónoma de México, Ciudad de México 04510, Mexico; genvazquez@ciencias.unam.mx; 4 Centre de Recherche sur l’Inflammation, Laboratoire d’Excellence Inflamex, Université Paris Cité, INSERM U1149, CNRS EMR8252, 75018 Paris, France; ulrich.blank@inserm.fr

**Keywords:** TGF-β, mast cells, secretion, CCL-2, TNF, RBL-2H3

## Abstract

TGF-β is a pleiotropic cytokine with both stimulatory and inhibitory effects on immune cells, depending on the microenvironmental context. It targets mast cells (MCs) in different physio-pathological conditions, such as inflammation and cancer. Besides acting as a potent chemoattractant for MCs, TGF-β regulates many other aspects of MCs’ physiology, including the secretion of many regulatory molecules. MCs secrete a variety of mediators, either pre-formed or newly synthesized, upon appropriate stimulation. CCL-2 chemokine and TNF cytokine act as potent chemoattractants for several immune cells and participate in the initiation of inflammatory responses by recruiting them to injured tissues. TGF-β regulates CCL-2 and TNF secretion in different cell types and under distinct cellular contexts. Here, we report that the treatment with TGF-β alone induces the secretion of both pre-formed and newly synthesized CCL-2 in the rat RBL-2H3 mast cells but not in mouse bone marrow-derived mast cells (BMMCs). TGF-β-induced CCL-2 secretion depends on rapid rearrangements of the actin cytoskeleton and, remarkably, on the early secretion of soluble TNF that triggers an autocrine TNF signaling. In conclusion, we found cooperation between TGF-β and TNF signaling pathways to promote the secretion of CCL-2 chemokine by MCs in a cell-context specific manner.

## 1. Introduction

Transforming Growth Factor β (TGF-β) is a widely expressed cytokine. It is also the prototype of a superfamily of growth factors with pleiotropic actions on cell proliferation, differentiation, survival, death, and migration. Its actions are important to regulate multiple biological processes, including tissue homeostasis, organ development, wound healing, and immune responses [1,2]. TGF-β exerts its actions by binding to a complex of transmembrane serine/threonine kinase receptors, TβRII and TβRI/ALK5 (Activin receptor-Like Kinase 5), to trigger the activation of Smad-dependent (canonical) and Smad-independent (non-canonical) signaling pathways. Activations of Mitogen-Activated Protein Kinases (MAPKs), Phosphatidyl-Inositol-3-Kinase (PI3K)/AKT, and Rho-GTPases are important processes involved in the non-canonical TGF-β signaling cascade. In the canonical pathway, activated Smad transcription factor complexes, including Smad2, Smad3, and/or Smad4, translocate to the nucleus and associate with other transcription factors or cofactors to regulate the expression of more than 500 different genes, in a cellular context-dependent manner [1,2,3].

TGF-β is an important regulatory cytokine that exerts both stimulatory and inhibitory effects on immune cells. TGF-β maintains immune tolerance via the regulation of lymphocyte function and also controls the initiation and resolution of inflammatory responses by regulating chemotaxis, activation, and survival of most leukocytes [4,5,6]. TGF-β also regulates leukocytes’ function in autoimmune diseases, where immunological tolerance to self-antigens is lost [7]. Intriguingly, TGF-β actions depend on the immune cell differentiation state and also on the presence of other cytokines.

Mast cells (MCs) are innate immune cells located in tissues that arise from the yolk sac during embryonic development and, in adults, are mostly derived from bone marrow precursors that migrate to all vascularized tissues of the body and finish their differentiation under the influence of locally produced mediators. Evidence shows that their origin and the signals from the tissue microenvironment determine MCs’ phenotype. Murine MCs are subclassified into two main subsets based on their tissue distribution and granule content: Connective tissue mast cells (CTMCs) originate from yolk sac-erythro-myeloid progenitors (EMPs), express tryptase, present high levels of the Mas-related G-protein receptor B2 (MRGPRB2), and are located around venules and nerve endings in most connective tissues (i.e., skin, adipose, trachea, tongue, esophagus, peritoneal cavity, and pleural cavity); and mucosal mast cells (MMCs) originate from fetal hematopoietic stem cells (HSCs), lack tryptase, and are located inside the epithelia of the gut and respiratory mucosa [8,9,10]. MCs secrete a variety of active compounds, either pre-formed or newly synthesized, that are liberated into their surrounding microenvironments upon appropriate stimulation. They include a wide range of biologically active cytokines, chemokines, lipid mediators, proteases, and biogenic amines [11,12]. MCs are an important source of cytokines, such as Tumor Necrosis Factor or TNF-α (TNF), interleukins such as IL-6, and also secrete certain chemokines, such as CCL-1, CCL-2, CCL-3, and CCL-4 [11,13].

MCs display a striking heterogeneity, generated by a complex interplay between different local microenvironmental signals and a differentiation program that decides their identity [9]. Depending on their tissue localization, MCs display differences in granule contents, cytokine expression patterns, and receptors; two hypothetical phenotypes called MC1 and MC2, related to pro-inflammatory and anti-inflammatory secretory profiles, respectively, have been described in a single population of MCs and are supported by MCs’ transcriptomic evidence [9,12,13]. Moreover, MCs display a trained memory that helps shape their function in their specific tissue microenvironment [14]. The content of MCs’ granules and the timing of their release, as well as their synthesis/replenishment, are key to determining MCs’ role in chronic tissue remodeling, allergic inflammation, fibrosis, and cancer progression. Therefore, it is important to investigate the molecular mechanisms that trigger each type of MCs degranulation/secretion pathway to direct pharmacological research toward disease-specific therapeutic targets, where MCs are involved [15].

TGF-β plays a multifaceted role in MCs’ development and survival depending on developmental stage, cytokine dose, and MCs’ subsets [16]. Many reports indicate that the TGF-β1 isoform can suppress the functions of diverse immune cells, including MCs, and it has been proposed that MC-derived TGF-β1 suppresses MCs’ functions in an autocrine or paracrine manner [17]. TGF-β is a potent chemoattractant for MCs and also regulates different aspects of MCs’ physiology, including the secretion of cytokines [13,16,17,18]. In the context of cancer, MCs seem to foster tumor progression by recruiting regulatory T lymphocytes (Tregs) through TGF-β signaling [13,16,19]. Furthermore, MCs-secreted TGF-β can reshape the tumor microenvironment, fostering fibrosis or desmoplasia by stimulating the proliferation and collagen production of fibroblasts, differentiating them into cancer-associated fibroblasts (CAFs), a critical event for metastasis, as well as it can promote local secretion of diverse cytokines and chemokines [13,20]. Thus, TGF-β regulates many aspects of MCs’ physiology and pathology by differentially regulating the behavior of MCs in a microenvironment context-dependent manner, and recently, it was reported that TGF-β is suitable to induce a mucosal gene signature [10]. Given that the primary culture of BMMCs usually consists of a population of heterogeneous cells not completely differentiated, whereas RBL-2H3 cells are considered as fully differentiated mucosal MCs, it would be interesting to investigate whether the TGF-β-induced cytokine secretion in RBL-2H3 cells also occurs in BMMCs (bone marrow-derived mast cells).

The secretion of pre-formed and newly formed mediators from MCs is a process that depends on multiple signaling pathways and also on MCs’ phenotype and context. TGF-β has an important role in human MCs development, mainly directing their differentiation and also maintaining their phenotype across tissues and diseases. TGF-β selectively reshapes MCs’ proinflammatory cytokine, chemokine, and growth factor production following immunoglobulin E (IgE) cross-linking. Furthermore, TGF-β enhances MCs’ lipid mediator production and regulates CCL-2 expression in a context-dependent manner [21]. Therefore, it would be important to investigate how TGF-β can impact differentially the behavior of each MC subtype, and whether the mechanisms involved include direct effects on genetic regulation, or whether, indirectly, TGF-β can cross-talk with other signaling pathways to modulate MCs’ physiology.

CCL-2 is a member of the CC chemokine family; it was identified as a potent chemotactic factor for monocytes, macrophages, natural killer cells, and MCs’ progenitors [22,23]. CCL-2 participates in the initiation of inflammatory responses by recruiting several immune cells to the allergic inflammation site or to the tumor microenvironment [24]. Recently, our group reported that tumor hypoxia induces CCL-2 secretion by primary BMMCs in a calcium-dependent manner [25]. Also, current evidence indicates that TNF can also stimulate the expression and secretion of CCL-2 [26,27]. TNF is a relevant mediator in regulating the inflammatory response, and MCs are an important source of TNF [11,13]. Intriguingly, TGF-β inhibits the secretion of TNF in IgE-sensitized BMMCs [28,29], whereas TGF-β is able to positively regulate CCL-2 production in different cell types, such as vascular smooth muscle cells [30,31], vascular endothelial cells [31,32], mesangial cells [33], and breast cancer cells [34].

The aim of this study was to investigate the production of CCL-2 by MCs in response to immunosuppressive cytokines such as TGF-β. To do this, we evaluated CCL-2 secretion by Enzyme-Linked ImmunoSorbent Assay (ELISA) in primary cultured BMMCs and in the RBL-2H3 mast cell line, and we also determined the TGF-β signaling pathways involved by using specific inhibitors of its canonical and non-canonical signaling pathways, as well as inhibitors of actin cytoskeleton remodeling. We observed that TGF-β induced secretion of CCL-2 and TNF in RBL-2H3 cells but, surprisingly, did not have the same effect in BMMCs. We revealed a TNF autocrine signaling loop generated by TGF-β signaling that promotes CCL-2 secretion in a MCs context-specific manner.

## 2. Results

### 2.1. TGF-β Stimulates the Secretion of Pre-Formed and de Novo-Synthesized CCL-2 Chemokine in RBL-2H3 Mast Cells

**Figure 1:**
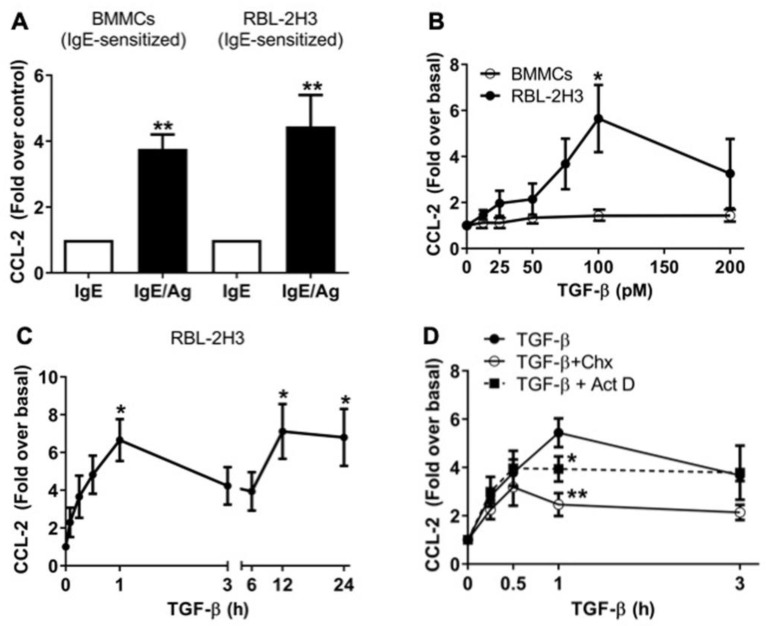
TGF-β stimulates the secretion of pre-formed and de novo synthesized CCL-2 chemokine in RBL-2H3 cells. (**A**) BMMCs and RBL-2H3 cells were sensitized overnight with 100 ng/mL IgE and then stimulated for 1 h with 27 ng/mL DNP-HAS (Ag), and CCL-2 secretion was evaluated in cell media by ELISA. Graphs show the mean ± SEM (*n* = 3, where *n* = number of data points), ** *p* ≤ 0.01 vs. control. (**B**) RBL-2H3 cells and IgE-sensitized BMMCs were stimulated with different concentrations of TGF-β as indicated. Conditioned media were collected, and CCL-2 levels were quantified by ELISA. Graphs show the mean ± SEM (*n* = 3), * *p* ≤ 0.05 vs. basal. (**C**) Time course of CCL-2 secretion after the addition of TGF-β. RBL-2H3 cells were stimulated with 100 pM TGF-β, and supernatants were collected at different times, and CCL-2 was detected by ELISA (*n* = 3), * *p* ≤ 0.05 vs. basal. (**D**) RBL-2H3 cells were pre-incubated for 30 min with 2 μg/mL ActD or 10 μg/ mL CHX before the stimulation for 1 h with 100 pM TGF-β. Media were collected, and CCL-2 secretion was evaluated by ELISA, * *p* ≤ 0.05, ** *p* ≤ 0.01 vs. TGF-β stimulated cells. Graphs show the mean ± SEM (*n* = 4).

MCs secrete diverse mediators and cytokines in response to the cross-linking of the high-affinity IgE receptor (FcεRI) by IgE/antigen (IgE/Ag) complexes [17], as well as in response to many other stimuli [11]. RBL-2H3 cells are commonly used as a model of MMCs, employed to analyze secretory events and cytokine production after FcεRI activation. To evaluate CCL-2 secretion after activation of the FcεRI receptor, BMMCs and RBL-2H3 cells were IgE-sensitized overnight and then stimulated for 1 h with 27 ng/mL dinitrophenol-coupled human serum albumin (DNP-HAS) as an Ag. As shown in Figure 1A, BMMCs showed a 3.7-fold increase, and RBL-2H3 cells showed a 4.4-fold increase in secreted CCL-2 in response to IgE/Ag/FcεRI axis activation.

We previously reported that TGF-β neither promotes MCs’ degranulation nor affects IgE/Ag-induced degranulation in BMMCs [35]; a similar result was observed in RBL-2H3 (Supplementary Figure S1). Nevertheless, TGF-β differentially influences the secretion of a variety of cytokines in MCs [36,37]; for instance, we observed that TGF-β primes MCs for higher VEGF production in response to IgE/Ag in BMMCs [35]. Thus, to test whether TGF-β regulates the secretion of CCL-2 chemokine in distinct context-dependent models of MCs, we first performed a dose–response curve using IgE-sensitized BMMCs. As it is shown in Figure 1B, we did not observe any significant effect on CCL-2 secretion when BMMCs were stimulated for 1 h with the indicated doses of TGF-β. In sharp contrast with BMMCs, stimulation of RBL-2H3 cells with 100 pM TGF-β increased CCL-2 release (5.6-fold over basal) (Figure 1B).

We then measured the secretion of this chemokine in time-course experiments in RBL-2H3 cells. We observed a 6.6-fold increase in CCL-2 release over the first hour of stimulation with 100 pM TGF-β, and interestingly, an accumulation of 7.1-fold over basal levels of CCL-2 was observed at 12 h, which was sustained until 24 h after the stimulation with TGF-β (Figure 1C). Remarkably, we observed a decrease in CCL-2 secretion between 1 and 3 h. To analyze whether this could be related to the endocytosis of the CCL-2/CCR2 complex, we performed the time-course experiment in the presence of the CCR2 receptor antagonist BMS CCR2 22, but that compound had no effect on CCL-2 secretion (Supplementary Figure S2). This suggests that secreted CCL-2 could be temporarily degraded extracellularly or endocytosed by an unknown mechanism before being secreted again between 6 and 12 h after TGF-β addition.

In MCs, cytokines are secreted mainly from two different intracellular pools, either pre-formed or newly synthesized. Secretion from both pools can be evaluated by adding actinomycin D (ActD) to inhibit de novo transcription or cycloheximide (CHX) to block de novo protein synthesis. Pretreatment of MCs with ActD or CHX diminished TGF-β-induced CCL-2 production detected after 1 h, but not that observed after 30 min of TGF-β addition (Figure 1D), suggesting that RBL-2H3 cells have a pool of pre-formed CCL-2 that is secreted shortly after stimulation (0.5 h). Intriguingly, de novo protein synthesis appears to be required to maintain continuous CCL-2 exocytosis (Figure 1D).

### 2.2. TGF-β/ALK5 Activates Canonical and Non-Canonical Pathways Implicated in CCL-2 Secretion in RBL-2H3 Cells

**Figure 2:**
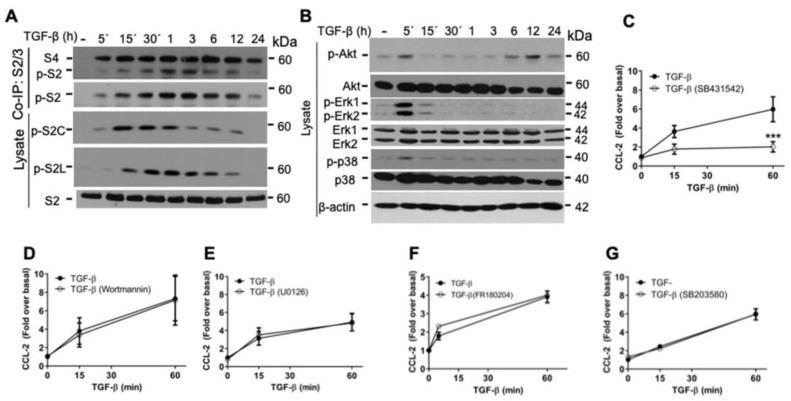
TGF-β activates canonical and non-canonical pathways to promote CCL-2 secretion in RBL-2H3 cells. (**A**,**B**) RBL-2H3 cells were stimulated with 100 pM TGF-β for the indicated times. Phosphorylation of representative proteins of the canonical (Smad2) (**A**) and non-canonical (AKT, ERK, p38) (**B**) signaling pathways was detected by WB (*n* = 3). RBL-2H3 cells were pre-incubated for 30 min with (**C**) 10 μM SB431542 (ALK5 inhibitor), (**D**) 3 nM Wortmannin (PI3K inhibitor), (**E**) 10 μM U0126 (MEK inhibitor), (**F**) 10 μM FR180204 (ERK1/2 inhibitor), or (**G**) 10 μM SB203580 (p38 inhibitor), and then cells were treated for 15 min and 1 h with 100 pM TGF-β. Cell media were collected at different times, and CCL-2 concentration was determined by ELISA. Graphs show the mean ± SEM of 3–5 experiments performed independently, *** *p* ≤ 0.001 vs. TGF-β stimulated cells.

TGF-β cytokine activates Smad-dependent (canonical) and Smad-independent (non-canonical) signaling cascades [1,2]. To analyze the canonical signaling pathways activated by TGF-β in RBL-2H3 cells, we evaluated the phosphorylation of Smad2 in the C-terminal SXS motif (p-S2C) and in the linker region (p-S2L) by Western blot (WB), as well as the association of Smad2 with Smad4 by co-immunoprecipitation (co-IP) and WB. We observed that TGF-β induced the phosphorylation of Smad2 (p-S2C and p-S2L) in a time-dependent manner and promoted an association of Smad2 and Smad4 proteins as early as 5 min; this association continued until 12–24 h after TGF-β stimulation (Figure 2A). Analyzing the TGF-β non-canonical signaling pathways, we observed that this cytokine caused an early phosphorylation of AKT (S473) at 5 min and a second pronounced peak at 6 h that lasted up to 24 h (Figure 2B). Likewise, we investigated the ERK1 and ERK2 phosphorylation and observed a sharp phosphorylation of ERK1/2 at 5 min after TGF-β treatment. Finally, we detected the phosphorylation of p38 at 5 min after TGF-β treatment and then returned to basal levels (Figure 2B).

To characterize the TGF-β signaling pathways involved in CCL-2 secretion, RBL-2H3 cells were pre-treated with different inhibitors before the treatment with TGF-β. Then, conditioned media were collected, and CCL-2 concentration was determined by ELISA. First, we tested the participation of the TGF-β receptor ALK5 in CCL-2 secretion by pre-treating RBL-2H3 cells with SB431542 (an inhibitor of Ser/Thr kinase activity of ALK5 receptor). In those conditions, the secretion of the pre-formed CCL-2 in response to TGF-β treatment for 1 h was decreased (Figure 2C). To analyze the role of the non-canonical pathways in CCL-2 secretion, cells were pre-incubated for 30 min with the inhibitors Wortmannin (PI3K), U0126 (MEK), FR180204 (ERK1/2), or SB203580 (p38) before the addition of TGF-β for 1 h, but those inhibitors had no effect on TGF-β-induced CCL-2 production (Figure 2D–G). Together, these results suggest that TGF-β-induced CCL-2 secretion observed in RBL-2H3 depends on ALK5 receptor, but it is independent of those non-canonical pathways.

Changes in the reorganization of the actin cytoskeleton are critical for a variety of cell activities, including secretion, and the inhibitors of actin polymerization can modulate mast cell degranulation [38]. Since we previously reported that the stimulation of MCs with TGF-β induces rapid actin polymerization/depolymerization cycles [39], we decided to also explore the participation of the Rho/Rho-associated kinase (ROCK)/F-actin axis, a non-canonical pathway that control the actin cytoskeleton dynamics that could be involved in TGF-β-induced CCL-2 secretion in RBL-2H3 cells.

### 2.3. TGF-β Induces Dynamic Re-Arrangements of the Actin Cytoskeleton Associated with CCL-2 Secretion in RBL-2H3 Cells

**Figure 3:**
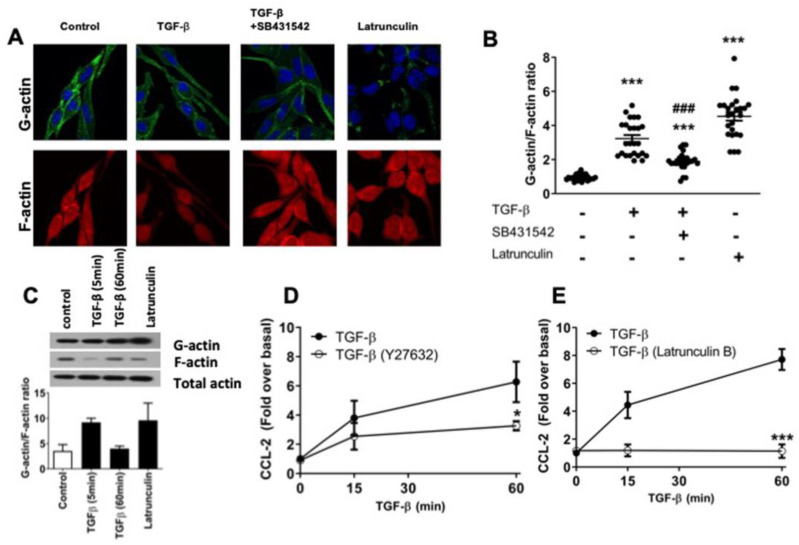
TGF-β-induced CCL-2 secretion is dependent on dynamic rearrangements of the actin-cytoskeleton in RBL-2H3 cells. (**A**,**B**) Fluorescence images from RBL-2H3 cells. Cells were incubated for 30 min in the absence (−) or presence (+) of 1 μM LatB or with 10 μM SB431542 before the stimulation for 5 min with 100 pM TGF-β. After treatment, cells were fixed and stained with Alexa-Fluor 594 conjugated Dnase I (G-actin) and FITC-phalloidin (F-actin). The scale bar represents 10 μm. (**B**) Quantification of G-actin/F-actin ratio in RBL-2H3 cells (*n* = 25 cells). *** *p* ≤ 0.001 vs. basal; ^###^ *p* ≤ 0.001 vs. TGF-β stimulated cells. (**C**) RBL-2H3 cells were pre-incubated for 30 min with 1 μM LatB or treated with 100 pM TGF-β for the indicated times. F-actin and G-actin fractions were obtained by centrifugation and analyzed by WB. Graphs show G-actin/F-actin ratio by densitometric analysis of WB data. RBL-2H3 cells were pre-incubated for 30 min with (**D**) 10 μM Y27632 (ROCK inhibitor) or (**E**) 1 μM LatB and then stimulated with 100 pM TGF-β for the indicated times. Cell media were collected at different times, and CCL-2 secretion was determined by ELISA. (**D**,**E**) Graphs show the mean ± SEM of 3–4 experiments performed independently, * *p* ≤ 0.05 (**D**), *** *p* ≤ 0.001 vs. TGF-β stimulated cells (**E**).

We analyzed the effect of TGF-β on actin cytoskeleton arrangements in RBL-2H3 cells. G-actin and F-actin pools were analyzed by immunofluorescence in RBL-2H3 cells (Figure 3A); then, the G-actin/F-actin ratio was measured (Figure 3B). TGF-β increased the G/F-actin ratio in RBL-2H3 cells (3.4-fold over basal), while the pretreatment with SB431542 (ALK5 inhibitor) prevented the F-actin depolymerization in response to TGFβ (Figure 3B). As a positive control, we used latrunculin B (LatB) that binds to monomeric actin, preventing actin polymerization. As expected, the G/F-actin ratio increased 4.9-fold in cells treated with LatB (Figure 3B). Furthermore, LatB increased G-actin levels, while it did not significantly affect the total amount of actin (Figure 3C). A lower proportion of F-actin was observed in cells stimulated for 5 min with TGF-β compared to 60 min (Figure 3C). In RBL-2H3 cells, ROCK has been described to be involved in actin remodeling downstream of TGF-β signaling [40]. Thus, we evaluated the involvement of the ROCK/F-actin axis in CCL-2 secretion and found that pre-incubation of RBL-2H3 cells with Y27632 (ROCK inhibitor) partially decreased TGF-β-induced CCL-2 secretion (Figure 3D). Then, we tested the participation of F-actin assembly in secretion. Cells were pre-treated with LatB and showed a reduced CCL-2 production in response to TGF-β (Figure 3E). This suggests that the exocytosis of CCL-2 regulated by TGF-β in RBL-2H3 also depends on the activation of the ROCK/F-actin axis.

### 2.4. TGF-β Stimulates the Secretion of Pre-Formed TNF via ERK1/2 Activation in RBL-2H3 Mast Cells

**Figure 4:**
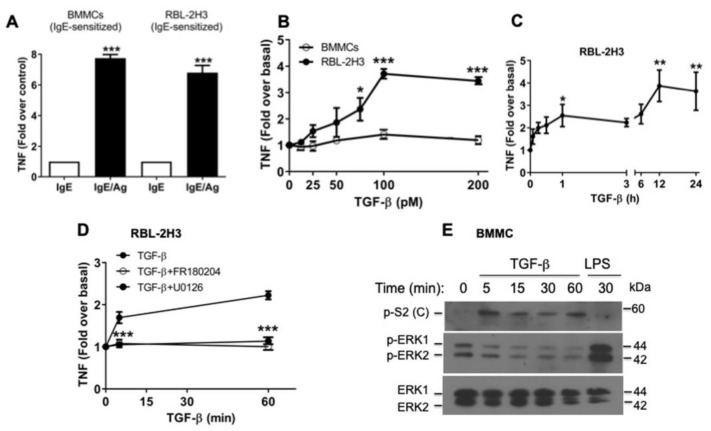
TGF-β stimulates the secretion of pre-formed TNF via ERK1/2 activation in RBL-2H3 mast cells. (**A**) BMMCs and RBL-2H3 mast cells were sensitized overnight with 100 ng/mL IgE and then stimulated for 1 h with 27 ng/mL DNP-HAS (Ag); TNF secretion was evaluated in cell media by ELISA. Graphs show the mean ± SEM of 3 experiments performed independently, *** *p* ≤ 0.01 vs. control. (**B**) RBL-2H3 cells and IgE-sensitized BMMCs were stimulated for 1 h with different concentrations of TGF-β. Conditioned media were collected, and TNF levels were quantified by ELISA. Graphs show the mean ± SEM of 3 experiments performed independently, * *p* ≤ 0.05 and *** *p* ≤ 0.001 vs. basal. (**C**) Time course of TNF secretion after the addition of TGF-β. RBL-2H3 cells were stimulated with 100 pM TGF-β for the indicated times, media were collected at different times, and TNF was detected by ELISA (*n* = 4), * *p* ≤ 0.05 and ** *p* ≤ 0.01 vs. basal. (**D**) RBL-2H3 cells were pre-incubated for 30 min with 10 μM U0126 (MEK inhibitor) or 10 μM FR180204 (ERK1/2 inhibitor), and then, cells were treated with 100 pM TGF-β for the indicated times. Cell media were collected at different times, and CCL-2 concentration was determined by ELISA. Graphs show the mean ± SEM of 3–5 experiments performed independently, *** *p* ≤ 0.001 vs. TGF-β stimulated cells. (**E**) BMMCs were sensitized overnight with 100 ng/mL IgE and then stimulated for 1 h with 100 pM TGF-β for the indicated times; LPS (500 ng/mL) treatment for 30 min was used as a control. Levels of p-Smad2(C), p-ERK1/2, and ERK1/2 were detected by Western blot (*n* = 2).

In some cells, there is evidence that CCL-2 secretion may depend on the action of distinct secreted mediators, such as TNF [26,27]. To analyze whether secreted TNF could play a role in CCL-2 production in MCs, we first determined the production of TNF after FcεRI receptor cross-linking in BMMCs and RBL-2H3 (Figure 4A). FcεRI triggered an increase in TNF secretion in both IgE-sensitized BMMCs (7.7-fold over control) and IgE-sensitized RBL-2H3 cells (6.8-fold over control), when cells were stimulated with 27 ng/mL DNP-HAS (Figure 4A).

Then, we decided to study the effect of TGF-β on TNF secretion from BMMCs and RBL-2H3. As expected, we observed that TGF-β did not induce TNF secretion from IgE-sensitized BMMCs (Figure 4B), but surprisingly, RBL-2H3 cells treated for 1 h with 100 pM TGF-β increased TNF release by 3.7-fold (Figure 4B). Next, we performed time-course experiments and measured TNF concentration in conditioned media of RBL-2H3 cells. As observed in Figure 4C, TNF release (2.5-fold over basal) occurred within the first hour of TGF-β stimulation; we then observed a second peak of TNF secretion at 12 h (3.8-fold) that lasted up to 24 h (Figure 4C).

Some studies indicate that ERK1/2 phosphorylation is required for the activation of the ADAM17/TACE protease, an enzyme responsible for the cleavage of pro-TNF and the release of mature TNF [41,42]. To evaluate the participation of ERK1/2 in TNF production in response to TGF-β, the inhibitors U0126 (MEK1) or FR180204 (ERK1/2) were added to the cells prior to TGF-β stimulus, and their effect on TGF-β-induced TNF secretion was evaluated. Figure 4D shows that U0126 and FR180204 compounds blocked soluble TNF secretion in response to TGF-β, whereas these inhibitors had no effect on early TGF-β-induced CCL-2 production (Figure 2F,G). Interestingly, although TGF-β signaling was able to induce a transient ERK1/2 phosphorylation in RBL-2H3 cells (Figure 2B), surprisingly, this was not observed in BMMCs (Figure 4E). These data demonstrate that TGF-β-induced TNF secretion in RBL-2H3 requires the activation of the ERK1/2 non-canonical pathway.

### 2.5. An Autocrine Regulator Loop Involving TNF Is Activated by TGF-β to Promote Late CCL-2 Secretion in RBL-2H3 Cells

**Figure 5:**
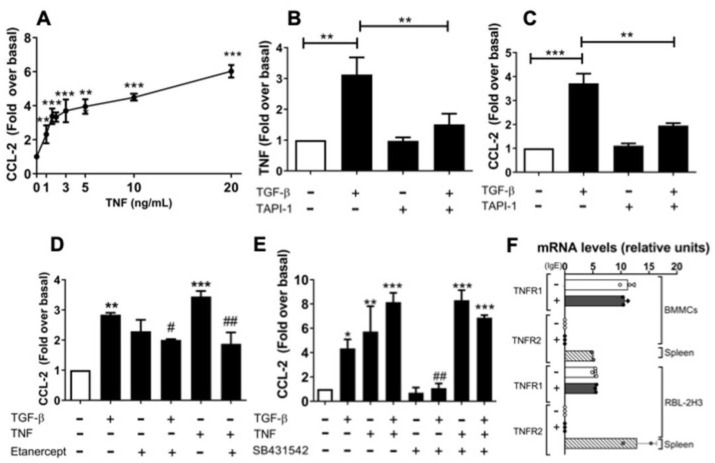
TGF-β-induced secreted TNF is required to promote full CCL-2 secretion in RBL-2H3 cells. (**A**) Dose-dependent secretion of CCL-2 induced by TNF. RBL-2H3 cells were stimulated with different concentrations of TNF for 1 h, media were collected at different times, and CCL-2 was detected by ELISA (*n* = 4), ** *p* ≤ 0.01 vs. basal; *** *p* ≤ 0.001 vs. basal. (**B**,**C**) RBL-2H3 cells were pre-incubated for 3 h in the absence (−) or presence (+) of 50 μM TAPI-1 before the addition of 100 pM TGF-β for 1 h, and then, the cell media were collected, and the concentrations of TNF (**B**) and CCL-2 (**C**) were detected by ELISA. Graphs show the mean ± SEM of 6–7 experiments performed independently, ** *p* ≤ 0.01; *** *p* ≤ 0.001. (**D**) RBL-2H3 cells were pre-incubated for 30 min with Etanercept and then stimulated for 1 h with 100 pM TGF-β or 3 ng/mL TNF. Cell-conditioned media were used to quantify CCL-2 levels by ELISA. Graphs show the mean ± SEM of 3 experiments performed independently, ** *p* ≤ 0.01 and *** *p* ≤ 0.001 vs. basal; ^#^ *p* ≤ 0.01 TGF-β vs. TGF-β plus Etanercept; ^##^ *p* ≤ 0.01 TNF vs. TNF plus Etanercept. (**E**) RBL-2H3 cells were pre-incubated for 30 min in the absence or presence of 10 μM SB431542 and then stimulated for 1 h with 65 pM TGF-β, 3 ng/mL TNF, or TGF-β + TNF. Then, cell media were collected, and CCL-2 secretion was quantified by ELISA. Graphs show the mean ± SEM of 3 experiments performed independently, * *p* ≤ 0.05; ** *p* ≤ 0.01; *** *p* ***1an ± S. basal; ^##^ *p* #asaln ± SEM of. TGF-β plus SB431542. (**F**) Total RNA was isolated from RBL-2H3, IgE-sensitized BMMCs, and from mouse spleen (as a control) to evaluate the presence of TNFR1 and TNFR2 mRNAs by RT-qPCR (*n* = 3).

Next, we tested the possible involvement of TNF in TGF-β-induced CCL-2 secretion in RBL-2H3 cells. First, we stimulated RBL-2H3 cells with increasing concentrations of exogenous recombinant TNF and detected a dose-dependent increase in CCL-2 secretion (Figure 5A). Since endogenous TNF is initially produced as a transmembrane precursor that is released by proteolytic cleavage catalyzed by ADAM17/TACE, we stimulated the cells with TGF-β in the absence or presence of TAPI-1 (TACE inhibitor). As expected, pre-incubation of cells with TAPI-1 partially blocked TGF-β-induced secretion of soluble TNF (Figure 5B) and CCL-2 (Figure 5C). These findings suggest that TGF-β-induced CCL-2 secretion may depend on autocrine TNF signals. We decided to further evaluate this hypothesis.

To corroborate the participation of TNF in TGF-β-induced CCL-2 secretion in RBL-2H3, we used Etanercept (ET), a TNF-blocking agent, which is a recombinant protein formed by part of the human TNF receptor coupled with the Fc portion of human IgG [43,44]. Considering that ET acts like a TNF decoy receptor, we decided to evaluate the capability of ET to inhibit TNF signaling. We measured CCL-2 secretion in RBL-2H3 cells pre-incubated with ET. CCL-2 secretion was partially suppressed by ET, when cells were treated with TGF-β or TNF, respectively (Figure 5D). We also observed that ET treatment enhances the basal levels of CCL-2, although it does not seem to interfere with TNF quantification by ELISA (Figure 5D).

Next, we analyzed the combination of TGF-β and TNF on CCL-2 secretion to determine whether both stimuli could be additive or synergistic. Stimulation with a suboptimal dose of 65 pM TGF-β induced a 4.3-fold increase in CCL-2 secretion, whereas a suboptimal dose of 3 ng/mL TNF induced a 5.7-fold increase in CCL-2 secretion. When cells were stimulated with both TGF-β plus TNF, there was a higher secretion of CCL-2 (8.1-fold), supporting an additive effect (Figure 5E). When TNF-treated cells were pre-incubated with SB431542, no differences in CCL-2 secretion were observed; meanwhile, when TGF-β plus TNF-stimulated cells were pre-incubated with SB431542, a slight decrease in CCL-2 secretion was observed (Figure 5E). These data support our previous conclusions regarding the effect of TGF-β on CCL-2 secretion, which was found to be partially dependent on soluble TNF secretion and autocrine TNF signaling. Finally, to analyze whether proper TNF receptors are expressed in MCs, expression of TNF receptor type 1 (TNFR1) and type 2 (TNFR2) was determined by RT-qPCR. Figure 5F shows that TNFR1 is expressed in both BMMCs and RBL-2H3 cells.

### 2.6. The Diversity of MCs’ Contexts Contributes to the Differential Secretion of Mediators in Response to TGF-β

**Figure 6:**
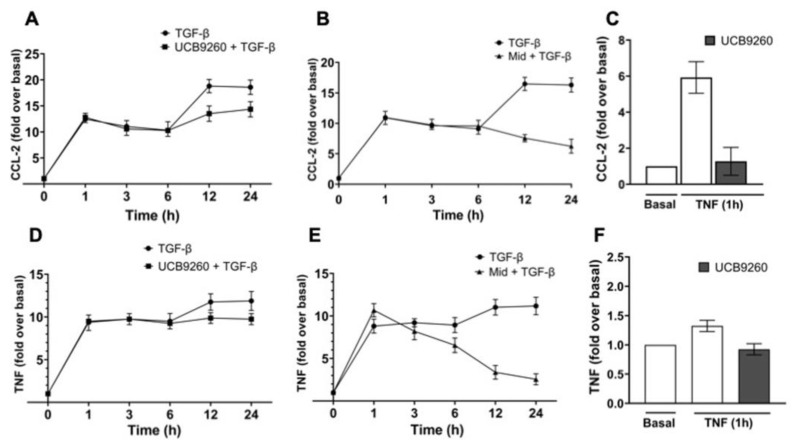
The late secretion of CCL-2 induced by TGF-β depends on the generation of an autocrine TNF loop and also on the MCs’ context. RBL-2H3 cells were pre-incubated for 30 min with 10 μM UCB-9260 (TNFR inhibitor) or 1 μM midostaurin (Mid) (KIT inhibitor), and then, cells were treated with 100 pM TGF-β (**A**,**B**,**D**,**E**) or 3 ng/mL TNF (**C**,**F**) for the indicated times. Cell media were collected at different times, and CCL-2 (**A**–**C**) and TNF (**D**–**F**) concentrations were determined by ELISA. Graphs show the mean ± SEM (*n* = 4).

To further determine whether secreted TNF might have any involvement in TGF-β-induced CCL-2 secretion, we evaluated the effect of UCB-9260, an inhibitor of TNF signaling, on TGF-β-induced CCL-2 and TNF secretion in RBL-2H3 cells (Figure 6). Cells were pre-incubated for 30 min with 10 μM UCB-9260 to inhibit TNF signaling, and then, TGF-β-induced secretion of CCL-2 (Figure 6A) and TNF (Figure 6C) was measured in time-course experiments. The data showed that the UCB-9260 compound mainly inhibited the late secretion of CCL-2 (Figure 6A,C) and TNF (Figure 6D,F) induced by TGF-β (Figure 6A,D) or TNF (Figure 6C,F). In addition, higher doses of UCB-9260 (50–100 μM) partially inhibited the early CCL-2 secretion induced by TGF-β (Supplementary Figure S3). Data suggest that TGF-β-induced CCL-2 secretion clearly depends on the generation of an autocrine TNF signaling loop in RBL-2H3 cells.

Finally, it has been shown that RBL-2H3 cells, unlike BMMCs, express a mutant version of the KIT receptor that is constitutively active [45]. To elucidate a possible role of KIT receptor on the capacity of TGF-β to induce CCL-2 secretion, we evaluated the early CCL-2 secretion (1 to 6 h) induced by TGF-β in the presence of midostaurin (KIT inhibitor). As observed in Figure 6B, TGF-β-dependent early CCL-2 secretion was not affected by the pre-treatment for 30 min with 1 μM midostaurin; whereas, unexpectedly, the second wave of CCL-2 secretion (12 to 24 h) induced by TGF-β was inhibited by midostaurin treatment. Furthermore, we observed that 30 min pre-treatment with midostaurin only blocked TGF-β-induced late TNF secretion but not early TNF secretion (Figure 6E). However, long-term treatment of RBL-2H3 mast cells (1 to 24 h) with 10 μM midostaurin also blocked TGF-β-induced early TNF secretion (Supplementary Figure S4). Data clearly suggest that the phenotypic and functional diversity of MCs appears to explain why MCs respond differentially to TGF-β signaling pathways.

## 3. Discussion

TGF-β is a pleiotropic cytokine that controls distinct aspects of the immune system, acting as a pro- or anti-inflammatory cytokine in a context-dependent manner [4,5,6]. The TGF-β signaling pathway has an important role as a modulator of immune cells’ behavior in pathologies such as inflammation, fibrosis, and carcinogenesis. In cancer, TGF-β can remodel TME and promote immune-evasive and pro-tumorigenic surroundings; thus, TGF-β is an attractive target for therapeutic intervention. Interestingly, some recent therapies are not designed to directly kill cancer cells but rather to limit their plasticity induced by signals such as TGF-β; hence, combinatorial approaches, such as immune checkpoint blockade therapy or chemotherapy, are now being combined with TGF-β inhibitors [46].

MCs are involved in immunological and non-immunological mechanisms in health and disease, in multiple organs and systems. The pathophysiology of MC-driven disorders is diverse, ranging from localized reactions to systemic disorders, such as inflammation, allergies, autoimmune diseases, and cancer [47]. In MCs, TGF-β has positive or negative effects on their physiology and survival, depending on the cellular microenvironment [13,16,17,18,48,49,50]. Also, TGF-β seems to inhibit late-stage MCs maturation [29]. However, recently, Meurer et al. [10] reported that TGF-β1/ALK5/SMAD2 and IL-3/ERK1/2 pathways converge to balance differentiation and proliferation of MCs. Intriguingly, TGF-β1 promotes MCs differentiation by the expression of several genes associated with a mucosal MC gene signature, whereas IL-3/ERK1/2 axis induces proliferation and an immature phenotype in BMMCs and also blocks TGF-β1 signaling to prevent late gene responses and differentiation [10].

MCs are a group of heterogeneous cells with different tissue localization and granule content; there is also species-specific variability, as human and murine MCs exhibit clear phenotypic and functional differences. Furthermore, mastocytosis is a disease where MCs can become transformed and may express mutant proteins, such as the tyrosine kinase receptor KIT, whose mutations produce a constitutively active protein. Some MC lines that express active mutant KIT receptors behave as MMCs and also exhibit a different physiology from primary MCs. Therefore, it becomes relevant to investigate whether each MC subtype responds differently to the same stimulus, such as the immunoregulatory cytokine TGF-β. Here, we analyzed the cytokine and chemokine secretion by MCs in response to TGF-β. We observed a differential secretion of CCL-2 and TNF between primary BMMCs and RBL-2H3 mucosal MCs. We observed that the stimulation of IgE-sensitized BMMCs with TGF-β did not induce CCL-2 or TNF secretion, but, surprisingly, TGF-β induced the secretion of CCL-2 and TNF in RBL-2H3 cells.

CCL-2 is a critical chemokine for the recruitment of immune cells to the sites of inflammation. The regulation of CCL-2 expression in response to TGF-β, through Smad-dependent and Smad-independent pathways, has been previously studied in other cell types; for instance, TGF-β induces CCL-2 production through Smad3 and PKCδ signaling in vascular endothelial cells [31,32]. In mesangial cells, TGF-β1 increases CCL-2 mRNA and protein in a time- and dose-dependent manner through pathways involving activation of ERK, p38, and ROS generation [33]. There is also evidence that CCL-2 gene expression can be upregulated in tumor cells by inflammatory mediators, such as TNF or TGF-β. The promoter region of the CCL-2 gene contains multiple cis-elements for different transcription factors. Specificity protein 1 (Sp1) regulates basal transcription of the CCL-2 gene by binding to a GC-box located in the proximal region of the 5′-untranslated region, whereas there are two NF-κB sites located in the distal region [51]. Additionally, TGF-β can induce CCL-2 gene expression by increasing the association of Smad3, EGR1, and RXRA transcription factors to the CCL-2 promoter region in human metastatic breast cancer cells [34]. Given that the regulation of TGF-β target genes is usually cell-type and cellular-context specific, we can hypothesize that, in the case of MCs, TGF-β may induce CCL-2 gene expression directly through Smad3 binding sites or via Smad3 recruitment by SP1 or indirectly through TNF secretion that can activate NF-κB to induce CCL-2 gene expression and de novo synthesis, and as a result, this may contribute to the late secretion of CCL-2. However, this hypothesis remains to be evaluated.

**Figure 7:**
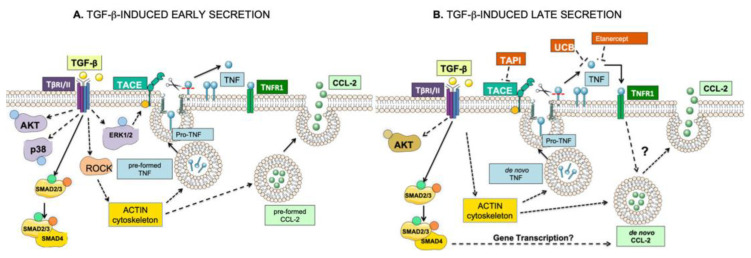
Model depicting the molecular mechanisms involved in the TGF-β-induced early and late secretion of CCL-2 in RBL-2H3 cells. (**A**) TGF-β activates ALK5-dependent canonical (Smads) and also non-canonical (AKT, ERK, p38, ROCK) pathways, causing rapid remodeling of the actin cytoskeleton, and, as a result, promotes the secretion of soluble TNF and CCL-2 from pre-formed pools. (**B**) Then, TGF-β cooperates with the soluble TNF to generate an autocrine TNF signaling loop involved in the late CCL-2 secretion from de novo-synthesized pools. The question marks (?) indicate unknown mechanisms.

Here, we report that the TGF-β-induced CCL-2 secretion in RBL-2H3 cell line is regulated mainly by ALK5 receptor-dependent signaling pathways, with the contribution of a non-canonical pathway that involves the dynamic re-organization of actin cytoskeleton and the Rho/ROCK signaling axis, which are essential events in CCL-2 secretion in response to TGF-β (Figure 7A). In support, we previously reported that TGF-β induces rapid actin polymerization/depolymerization cycles in BMMCs to promote migration [39]. Others have reported that TGF-β can induce a rapid actin reorganization via Rho GTPase-dependent pathways in RBL-2H3 cells, whereas long-term effects require the cooperation between the ALK5/Smad canonical and Rho GTPase signaling pathways [40].

We also found that RBL-2H3 cells secrete an important fraction of CCL-2 from a pool of pre-formed chemokine that is rapidly released after TGF-β stimulation (during the first 0.5 to 1 h), which overlaps in time with a late CCL-2 secretion from a de novo-synthesized pool that appears to depend on gene transcription and de novo protein synthesis; however, the specific molecular mechanisms involved may be varied and remain to be investigated. For instance, previous reports showed that TGF-β1 stimulated CCL-2 expression in mesangial cells, in part, by increasing mRNA stability [33], whereas it enhanced the binding of Smad4 to the CCL-2 promoter in endothelial cells [31]. Interestingly, TGF-β was able to inhibit CCL-2 expression in macrophages through an antagonistic effect of Smad3 on AP-1 activity [52]. All these evidences suggest that the mechanism of TGF-β to regulate CCL-2 gene expression is cell type-dependent.

Regarding TNF secretion in IgE-sensitized BMMCs, TGF-β inhibits the secretion of this cytokine, as reported previously [28]. However, unexpectedly, we observed here that TGF-β induces the secretion of TNF in RBL-2H3 cells, and this soluble TNF partially mediates TGF-β-induced CCL-2 secretion (Figure 7B). In support, emerging evidence has demonstrated that TNF is able to induce the expression and secretion of CCL-2. For example, TNF was shown to increase CCL-2 mRNA and protein secretion in endothelial cells [53], and the blockage of TNF actions reduces CCL-2 levels in patients with rheumatoid arthritis [26,54]. Here, we showed that TNF-induced CCL-2 secretion was dependent on dose in RBL-2H3. Furthermore, suboptimal doses of both stimuli, i.e., TGF-β plus TNF, seem to cooperate to regulate CCL-2 chemokine secretion. Our group and others have shown that TNF is secreted from two different intracellular pools in BMMCs: a preformed pool and a de novo-synthesized pool. Secretion of pre-formed TNF depends on IKK and ERK1/2 activation, whereas secretion of de novo-synthesized TNF relies on the participation of NF-κB and TNF mRNA stabilization [55,56,57,58]. The synergistic effect of TGF-β and TNF has also been previously observed in astrocytes, as their co-stimulation with TGF-β and TNF increased the expression of NOS-2 [59]. Likewise, TGF-β and TNF acted synergistically to increase the secretion of IL-6 in an intestinal epithelial cell line [60]. Our results suggest that TNF and CCL-2 secretion are intimately related and collaborate in the process connecting tissue damage to immune cell attraction. For example, in human skin-derived MCs and human freshly isolated MCs, activation of MRGPRX2 receptor (the human homologous of the murine MRGPRB2) leads to the production of TNF and CCL-2, indicating that synergistic actions of those cytokines are present in inflammation associated with pseudoallergic reactions [61]. Also, TNF leads to the secretion of CCL-2 by mesenchymal stem/stromal cells (MSCs) in invasive ductal carcinoma, and this was related to an important migration of monocytic cells [62]. In that study, the authors suggested that this TNF/CCL-2 axis could yield pro-cancerous myeloid infiltrates in breast tumors.

RBL-2H3 cells have been widely used as a MCs’ model, as they are easily grown in culture, are responsive to FcεRI-mediated cross-linking, can be genetically manipulated, and provide advantages over primary MCs, particularly for molecular studies [45]. Numerous studies indicate similar mechanisms of exocytosis of BMMCs and RBL-2H3 cells. Not only are the fusion machineries used by BMMCs and RBL-2H3 similar, but also the accessory proteins that regulate SNARE function, secretory granules motility, or secretory granules size. Moreover, FcεRI-activated BMMCs and RBL-2H3 cells revealed similar actin cytoskeleton remodeling linked with BMMCs- or RBL-2H3-stimulated exocytosis [45]. Furthermore, immortalized human or murine MCs’ lines have been widely used in research to study the molecular and cellular mechanisms involved in allergic diseases and also to develop effective allergy therapy. However, their use has its drawbacks, since every MC line can show some differences in their phenotypes and also behave differently from each other and from primary cells [63]. Therefore, it becomes important to carry out comparative experiments in both MCs’ lines and primary cells and, when possible, also use in vivo models.

Here, we observed that both BMMCs and RBL-2H3 cells express TNFR1 receptor but not TNFR2 (Figure 5F), as shown previously [64]. Interestingly, our data show that TGF-β induces the secretion of pre-formed CCL-2 and TNF mediators in RBL-2H3 cells but not in BMMCs; also, TGF-β promotes CCL-2 de novo synthesis for late secretion. In addition, TGF-β induces the secretion of TNF in RBL-2H3, and probably also its de novo synthesis. Strikingly, this secreted TNF induced by TGF-β was able to activate an autocrine TNF signaling and cooperate with the TGF-β pathway to promote full CCL-2 secretion (see model in Figure 7). TAPI inhibits TNF processing by blocking ADAM17/TACE activity, but this shedding protease may also lead to different cytokine release from cell membrane-precursor proteins, as for example, EGF and IL-6 [65,66]. This suggests a potential participation of other cytokines besides TNF in the observed CCL-2 secretion; however, the use of UCB-9260, a selective TNF signaling inhibitor that binds and stabilizes an asymmetric form of the TNF trimer, confirms the participation of TNF signaling downstream of the TGF-β pathway. Therefore, it is clear that TGF-β activates an autocrine TNF regulator loop linking TNF and CCL-2 secretion in RBL-2H3 mast cells (see model in Figure 7). Altogether, our results strongly suggest that maximal TGF-β-induced CCL-2 secretion involves several overlapping mechanisms that remain to be elucidated in detail.

Alterations in the immune system homeostasis lead to the development of multiple diseases, including inflammation, autoimmune diseases, and even carcinogenesis. MCs are implicated in resolving tissue inflammation, reshaping tissue microenvironments, and influencing host behavior [67]. In such conditions, the production of immunoregulatory cytokines is increased, such as TGF-β, whose actions and molecular mechanisms may vary according to cell context and tissue microenvironment. It is common that TGF-β acts directly or through a cross-talk with other signaling pathways to regulate immune cells, mainly in pathological conditions, such as inflammation, fibrosis, and cancer [68]. For instance, there is evidence that once MCs are recruited to TME, they can enhance some fibroblast functions, such as contracting collagen networks, through activation of SCF/c-Kit, IL-1α, and TNF signaling pathways. MCs may also secrete diverse compounds to foster tumor fibrosis, such as histamine, tryptase, and TGF-β, which activate fibroblasts to produce collagen. CCL-2 attracts fibrocytes to distinct injuries, and interestingly, the interplay among TGF-β, CCL-2, and IL-13 has been observed in pulmonary fibrosis, suggesting that it may also play a role in tumor fibrosis [13,69].

BMMCs and RBL-2H3 possess similar and different phenotypic characteristics, which could explain their differential responses to TGF-β. Moreover, it is important to consider that RBL-2H3 cells were generated from an RBL cell clone obtained from a rat with chemically induced leukemia, and they express a mutant version of the KIT receptor that is constitutively active [45]. Interestingly, only the late CCL-2 secretion induced by TGF-β was inhibited by midostaurin, a KIT inhibitor. In addition, the cancer context adds complexity as the leukemic mast cells respond differentially to TGF-β. Therefore, the evidence provided here opens the possibility of studying in detail how the diversity of MCs’ contexts determines the secretion of particular mediators in response to diverse stimuli, including cytokines such as TGF-β.

## 4. Materials and Methods

### 4.1. Reagents and Antibodies

Recombinant human TGF-β1 (TGF-β) and recombinant murine TNF-α (TNF) were purchased from PeproTech/ThermoFisher Scientific (Rocky Hill, NJ, USA). Culture reagents and media were obtained from Gibco/Life Technologies/ThermoFisher Scientific (Rocky Hill, NJ, USA), and Fetal Bovine Serum (FBS) was from BioWest (Bradenton, FL, USA). Etanercept (Enbrel^®^) was from Pfizer (New York, NY, USA). The inhibitors Wortmannin, ActD, and CHX were obtained from Merck/MilliporeSigma (Burlington, MA, USA); SB431542, SB202580, U0126, FR180204, and BMSCCR2 were obtained from Tocris Bioscience (Bristol, UK); Y27632 and LatB were from Calbiochem-Merck/Millipore (Burlington, MA, USA); UCB-9260 and Midostaurin were from MedChemExpress (Monmount Junction, NJ, USA). TAPI-1 and anti-β-actin antibodies were obtained from Santa Cruz Biotechnology (Dallas, TX, USA). Lipopolysaccharide (LPS) (L8274) was from Merck/MilliporeSigma (Burlington, MA, USA). FITC-conjugated phalloidin and Alexa-Fluor^TM^ 594 Dnase I were from Molecular Probes/ThermoFisher Scientific (Rocky Hill, NJ, USA). The following primary antibodies were from Cell Signaling Technology (Danvers, MA, USA): anti-pERK1/2, anti-p-Smad2, anti-p-AKT, anti-p-p38, anti-ERK1/2, anti-AKT, anti-p38, anti-Smad2/3, and anti-Smad4. The Alexa Fluor 488-conjugated anti-goat secondary antibody and both HRP-conjugated anti-rabbit and anti-mouse secondary antibodies were from Thermo Fisher Scientific (Rocky Hill, NJ, USA).

### 4.2. Cell Culture

RBL-2H3 cells were obtained from ATCC (CRL-2256) (Manassas, VA, USA) and were cultured at pH 7.4 in Dulbecco’s modified Eagle’s medium (DMEM) supplemented with 10% FBS, 3.5 mg/mL sodium bicarbonate, 1mM of sodium pyruvate, 100 IU/mL penicillin, 100 µg/mL streptomycin, and 1X non-essential amino acids (NEAAs). Cells were maintained at 37 °C in a humidified atmosphere of 5% CO_2_. For experiments, cells were dissociated with 0.25% Trypsin and replated, grown to confluence, and used ~24 h after seeding. Confluent cells were serum-starved for 2 h and then incubated with TGF-β or TNF, as indicated.

Primary BMMCs were obtained from bone marrow of C57BL/6J mice (stock no. 000664) from The Jackson Laboratory (Bar Harbor, ME, USA), as previously described [11,39,70]. Mice were euthanized following experimental procedures approved by our Institutional Committee for the Care and Use of Laboratory Animals (CICUAL protocols 0137-15; 0074-13) and following the ARRIVE guidelines for animal research. BMMCs were cultured for four weeks in RPMI 1640 medium at pH 7.4, supplemented with 10% FBS, 20 ng/mL IL-3, 50 μM β-mercaptoethanol, 25 mM HEPES, 1 mM pyruvate, 100 IU/mL penicillin, 100 µg/mL streptomycin, and 1X NEAA. When indicated, BMMCs and RBL-2H3 were sensitized for 24 h with 100 ng/mL IgE plus anti-Dinitrophenyl (mouse monoclonal SPE-7 clone, Merck/Millipore (Burlington, MA, USA), before stimulation for 1 h with 27 ng/mL DNP-HAS (Merck/Millipore (Burlington, MA, USA)), in the absence or presence of 100 pM TGF-β for the indicated times.

### 4.3. ELISA

Conditioned media from BMMCs and RBL-2H3 cells were collected after treatment with TGF-β or TNF. Each aliquot was centrifuged for 10 min at 360× *g* at 4 °C to discard cells, and then, the samples were used to evaluate CCL-2 or TNF protein levels. In some cases, prior to TGF-β treatment, cells were pre-treated for 30 min with different inhibitors, such as 2 μg/mL ActD (RNA pol II inhibitor), 10 μg/mL CHX (protein synthesis inhibitor), 10 μM SB431542 (ALK5 inhibitor), 10 µM Y27632 (ROCK inhibitor), 3 nM Wortmannin (PI3K inhibitor), 10 μM U0126 (MEK inhibitor), 10 μM FR180204 (ERK1/2 inhibitor), 10 μM SB202190 (p38 inhibitor), 10 µM SP600125 (JNK inhibitor), or 1 μM LatB (actin cytoskeleton disrupter), whereas cells were pre-incubated for 3 h with 50 μM TAPI-1 (TACE inhibitor) before treatment for 1 h with 100 pM TGF-β. In some experiments, before cells were treated for 1 h with 3 ng/mL TNF or 100 pM TGF-β, they were pre-treated for 30 min with 0.1 µg/mL Etanercept that acts as a TNF antagonist [43]. Secreted CCL-2 or TNF levels were quantified using cytokine-specific ELISA kits (900-K126) and (900-K54), respectively, and following the manufacturer’s instructions. ELISA kits were obtained from PeproTech/ThermoFisher Scientific (Rocky Hill, NJ, USA).

### 4.4. Immunofluorescence

Adherent RBL-2H3 cells were grown on 12 mm glass coverslips. After treatment, cells were fixed for 20 min with 4% paraformaldehyde in PBS at room temperature (RT) and washed with PBS. Then, cells were incubated for 10 min with 50 mM NH_4_Cl at RT and washed again with PBS. Non-specific binding was blocked with the blocking solution (3% bovine serum albumin, 5% donkey serum, and 0.01% Tween-20 in PBS). Primary and secondary antibodies were diluted in blocking solution. Cells were incubated overnight at 4 °C with primary antibody anti-TNF (1:100), with FITC-conjugated phalloidin (1:250) or with Alexa-Fluor 594 conjugated Dnase I (1:500). Next, nuclei were stained for 5 min with DAPI at RT. Cell images were acquired using a confocal microscope (Carl-Zeiss LSM-800, Oberkochen, Germany), and Zen lite V 2.4 software was used for image acquisition. Images were analyzed with the ImageJ software (2.0.0-rc-44).

### 4.5. F-Actin/G-Actin Separation

Total F-actin and G-actin fractions were obtained following a modified version of a protocol previously described [71]. Briefly, treated cells were washed once with PBS before cell lysis for 10 min at 37 °C with actin stabilization buffer (50 mM PIPES pH 6.9, 50 mM NaCl, 5 mM MgCl_2_, 5 mM EGTA, 2 mM ATP, 5% glycerol, 0.1% Nonidet P-40, 0.1% Triton X-100, 0.1% Tween 20, 0.1% β-mercaptoethanol) and protease and phosphatase inhibitors (1 mM PMSF, 1 µg/mL leupeptin, 1 µg/mL pepstatin, and 1 mM sodium orthovanadate). Cells were collected into microtubes and centrifuged for 3 min at 300× *g* at RT to remove insoluble particles. Protein concentration was determined using the Bio-Rad protein assay. Samples with equivalent protein concentration from each cell lysate were centrifuged for 75 min at 16,000× *g* at 4 °C. Supernatant containing G-actin was recovered, and the pellet containing F-actin was incubated with 1 μM Lat B in cold distilled water and kept on ice for 45 min to dissolve F-actin. G-actin and F-actin fractions were separated on 12% SDS-PAGE gels and then analyzed by WB. LatB interacts with G-actin in a 1:1 ratio to inhibit F-actin polymerization in vitro (IC_50_ = ~100 nM) [72].

### 4.6. Western Blot and Immunoprecipitation

RBL-2H3 cells were washed with ice cold PBS and then lysed in RIPA buffer (50 mM Tris-HCl pH 7.4, 150 mM NaCl, 1 mM EDTA, 0.5% Triton X-100, 0.1% SDS, 0.5% Sodium Deoxycholate, 1% Nonidet P-40), plus proteases and phosphatases inhibitors (1 mM NaF, 1 mM sodium orthovanadate, 1mM NaPPi, 1 mM PMSF, 1 µg/mL Trypsin inhibitor, 1 µg/mL Leupeptin, 1 µg/mL pepstatin A, 1 µg/mL benzamidine, and 10 mM β-glycerophosphate). Protein concentration was determined using the Bio-Rad protein assay. Equal amounts of protein were separated on an 8% SDS-PAGE gel, transferred onto polyvinylidene difluoride (PVDF) membrane, and subjected to immunoblotting. For immunoprecipitation assays, cells were washed with ice cold PBS and then lysed in modified lysis buffer (1% NP-40, 2 mM phenylmethylsulfonyl fluoride (PMSF), 5 mM sodium pyrophosphate, 60 mM octyl-β-glucoside, 50 mM NaF, 1 mM sodium orthovanadate, 10 μg/mL of aprotinin, 2 μg/mL pepstatin, and 2 μg/mL leupeptin). After protein quantification, 75 μg of protein was used for loading controls, and 300 μg of protein was used for the immunoprecipitation (IP) assays. Immunoprecipitated protein complexes were boiled, resolved in a 10% SDS-PAGE gel, and transferred to a PVDF membrane. Membranes were blocked for 1 h with 5% non-fat milk and washed twice with TBS-T before the addition of specific antibodies for WB detection using Immobilon Western Chemiluminescent HRP substrate (Merck/Millipore, Burlington, MA, USA). For BMMCs, 2 × 10^6^ cells were lysed in 200 μL of Laemmli sample buffer plus 5 mM sodium orthovanadate and 10% β-mercaptoethanol, boiled for 15 min, and stored at −70 °C; for WB, gels were loaded with 30 μL of protein sample per well.

### 4.7. Total RNA and RT-qPCR

Total RNA was extracted from two million cells (BMMCs or RBL-2H3) using TRI-reagent (Merck/MilliporeSigma, Burlington, MA, USA) according to the manufacturer’s instructions, and cDNA was generated using the Superscript First-strand Synthesis System (Life Technologies/ThermoFisher Scientific, Rocky Hill, NJ, USA). The mRNA levels of TNFR1 (mouse and rat), TNFR2 (mouse and rat), and GAPDH (control) were evaluated by qPCR using RealQ Plus 2x Master Mix (Cat. No. A323402) and the Qiagen Rotor Gene Q Real-Time System. Following primers were used for amplification of mTNFR1 Fw: 5′-GCTGTTGCCCCTGGTTATCT-3′ and Rv: 5′-ATGGAGTAGACTTCGGGCCT-3′; rTNFR1 Fw: 5′-TCGGGCTTACTGGATACGAGA-3′ and Rv: 5′-GGGTGTATCCCCATCAGCAG-3′; mTNFR2 Fw: 5′-AGGGTCTTTAGCCTCTTGCC-3′ and Rv: 5′-TTCACGATGCAGGTGACGTT-3′; rTNFR2 Fw: 5′-CAGGGACGTTCTCTGACACC-3′ and Rv: 5′-AGCAGTTCGCCAGTCCTAAC-3′; and GAPDH Fw: 5′-ATTGTGGAAGGGCTCATGAC-3′ and Rv: 5′-AGTGGATGCAGGGATGATGT-3′, and following the program: 95 °C × 2 min; 40 cycles at 95 °C × 5 s; and 60 °C × 30 s. The relative quantification of PCR products (qPCR) was performed using the 2^−∆∆CT^ method, and data were normalized to GAPDH expression.

### 4.8. Statistical Analysis

Data were expressed as the mean ± S.E.M of at least three independent experiments. Data were analyzed using one-way ANOVA and the Student–Newman–Keuls multiple comparison as a post hoc test. A *p* < 0.05 was considered significant. All analyses were performed with GraphPad Prism v.5.

## Data Availability

Data will be made available on request.

## Supplementary Materials

The following supporting information can be downloaded at https://www.mdpi.com/article/10.3390/ijms26094263/s1.

## Abbreviations

ActD, Actinomycin D; Ag, Antigen; ALK5, Activin receptor-Like Kinase 5; BMMCs, Bone Marrow-derived Mast Cells; CHX, Cycloheximide; CTMCs, Connective Tissue Mast Cells; ELISA, Enzyme-Linked ImmunoSorbent Assay; ET, Etanercept; IgE, Immunoglobulin E; LatB, Latrunculin B; MAPKs, Mitogen-Activated Protein Kinases; MCs, Mast Cells; MMCs, Mucosal Mast Cells; PI3K, Phosphatidyl-Inositol-3-Kinase; ROCK, Rho-associated Kinase; TGF-β, Transforming Growth Factor-β; TME, Tumor MicroEnvironment; TNF, Tumor Necrosis Factor; WB, Western blot.

## References

[1] Massagué J. (2014). TGF-beta signalling in context. Nat. Rev. Mol. Cell Biol..

[2] David C.J., Massagué J. (2018). Contextual determinants of TGFβ action in development, immunity and cancer. Nat. Rev. Mol. Cell Biol..

[3] Tecalco-Cruz A.C., Ríos-López D.G., Vázquez-Victorio G., Rosales-Alvarez R.E., Macías-Silva M. (2018). Transcriptional Cofactors Ski and SnoN are Major Regulators of TGF-beta/Smad Signaling Pathway in Health and Disease. Signal Transduct. Target. Ther..

[4] Li M.O., Wan Y.Y., Sanjabi S., Robertson A.-K.L., Flavell R.A. (2006). Transforming Growth Factor-Β Regulation of Immune Responses. Annu. Rev. Immunol..

[5] Batlle E., Massagué J. (2019). Transforming Growth Factor-β Signaling in Immunity and Cancer. Immunity.

[6] Chen W., Ten Dijke P. (2016). Immunoregulation by members of the TGFβ superfamily. Nat. Rev. Immunol..

[7] Li M.O., Flavell R.A. (2006). TGF-β, T-cell tolerance and immunotherapy of autoimmune diseases and cancer. Expert. Rev. Clin. Immunol..

[8] Li Z., Liu S., Xu J., Zhang X., Han D., Liu J., Xia M., Yi L., Shen Q., Xu S. (2018). Adult Connective Tissue-Resident Mast Cells Originate from Late Erythro-Myeloid Progenitors. Immunity.

[9] Tauber M., Basso L., Martin J., Bostan L., Pinto M.M., Thierry G.R., Houmadi R., Serhan N., Loste A., Blériot C. (2023). Landscape of mast cell populations across organs in mice and humans. J. Exp. Med..

[10] Meurer S.K., Bronneberg G., Penners C., Kauffmann M., Braunschweig T., Liedtke C., Huber M., Weiskirchen R. (2025). TGFB1 induces mucosal mast cell genes and is negatively regulated by the IL-3/ERK1/2 axis. Cell Commun. Signal.

[11] Blank U., Madera-Salcedo I.K., Danelli L., Claver J., Tiwari N., Sanchez-Miranda E., Vázquez-Victorio G., Ramírez-Valadez K.A., Macías-Silva M., González-Espinosa C. (2014). Vesicular trafficking and signaling for cytokine and chemokine secretion in mast cells. Front. Immunol..

[12] Solimando A.G., Desantis V., Ribatti D. (2022). Mast Cells and Interleukins. Int. J. Mol. Sci..

[13] Segura-Villalobos D., Ramírez-Moreno I.G., Martínez-Aguilar M., Ibarra-Sánchez A., Muñoz-Bello J.O., Anaya-Rubio I., Padilla A., Macías-Silva M., Lizano M., González-Espinosa C. (2022). Mast Cell–Tumor Interactions: Molecular Mechanisms of Recruitment, Intratumoral Communication and Potential Therapeutic Targets for Tumor Growth. Cells.

[14] Frossi B., Mion F., Sibilano R., Danelli L., Pucillo C.E.M. (2018). Is it time for a new classification of mast cells? What do we know about mast cell heterogeneity?. Immunol. Rev..

[15] Aitella E., Romano C., Ginaldi L., Cozzolino D. (2025). Mast Cells at the Crossroads of Hypersensitivity Reactions and Neurogenic Inflammation. Int. J. Mol. Sc..

[16] Haque T.T., Frischmeyer-Guerrerio P.A. (2022). The Role of TGFβ and Other Cytokines in Regulating Mast Cell Functions in Allergic Inflammation. Int. J. Mol. Sci..

[17] Mukai K., Tsai M., Saito H., Galli S.J. (2018). Mast cells as sources of cytokines, chemokines, and growth factors. Immunol. Rev..

[18] Kelly A., Houston S.A., Sherwood E., Casulli J., Travis M.A. (2017). Regulation of Innate and Adaptive Immunity by TGFβ. Adv. Immunol..

[19] Zhao Y.B., Yang S.H., Shen J., Deng K., Li Q., Wang Y., Cui W., Ye H. (2020). Interaction between regulatory T cells and mast cells via IL-9 and TGF-β production. Oncol. Lett..

[20] Murray L.A., Argentieri R.L., Farrell F.X., Bracht M., Sheng H., Whitaker B., Beck H., Tsui P., Cochlin K., Evanoff H.L. (2008). Hyper-responsiveness of IPF/UIP fibroblasts: Interplay between TGFβ1, IL-13 and CCL2. Int. J. Biochem. Cell Biol..

[21] Derakhshan T., Hollers E., Perniss A., Ryan T., McGill A., Hacker J., Bergmark R.W., Bhattacharyya N., Lee S.E., Maxfield A.Z. (2025). Human intraepithelial mast cell differentiation and effector function are directed by TGF-β signaling. J. Clin. Investig..

[22] Oliveira S.H., Lukacs N.W. (2001). Stem cell factor and IgE- stimulated murine mast cells produce chemokines (CCL2, CCL17, CCL22) and express chemokine receptors. Inflamm. Res..

[23] Collington S.J., Hallgren J., Pease J.E., Jones T.G., Rollins B.J., West-wick J., Austen K.F., Williams T.J., Gurish M.F., Weller C.L. (2010). The role of the CCL2/CCR2 axis in mouse mast cell migration in vitro and in vivo. J. Immunol..

[24] Jin J., Lin J., Xu A., Lou J., Qian C., Li X., Wang Y., Yu W., Tao H. (2021). CCL2: An Important Mediator Between Tumor Cells and Host Cells in Tumor Microenvironment. Front. Oncol..

[25] Ramírez-Moreno I.G., Ibarra-Sánchez A., Castillo-Arellano J.I., Blank U., González-Espinosa C. (2020). Mast Cells Localize in Hypoxic Zones of Tumors and Secrete CCL-2 under Hypoxia through Activation of L-Type Calcium Channels. J. Immunol..

[26] Harkness K.A., Sussman J.D., Davies-Jones G.A.B., Greenwood J., Woodroofe M.N. (2003). Cytokine regulation of MCP-1 expression in brain and retinal microvascular endothelial cells. J. Neuroimmunol..

[27] Chui R., Dorovini-Zis K. (2010). Regulation of CCL2 and CCL3 expression in human brain endothelial cells by cytokines and lipopolysaccharide. J. Neuroinflamm..

[28] Bissonnette E.Y., Enciso J.A., Refus A.D. (1997). TGF-β1 Inhibits the Release of Histamine and Tumor Necrosis Factor-α from Mast Cells Through an Autocrine Pathway. Am. J. Respir. Cell Mol. Biol..

[29] Kashyap M., Bailey D.P., Gomez G., Rivera J., Huff T.F., Ryan J.J. (2005). TGFB1 inhibits late-stage mast cell maturation. Exp. Hematol..

[30] Takeshita A., Chen Y., Watanabe A., Kitano S., Hanasawa S. (1995). TGF-beta induces expression of monocyte chemoattractant JE/monocyte chemoattractant protein 1 via transcriptional factor AP-1 induced by protein kinase in osteoblastic cells. J. Immunol..

[31] Ma J., Wang Q., Fei T., Han J.J., Chen Y. (2007). MCP-1 mediates TGF-beta–induced angiogenesis by stimulating vascular smooth muscle cell migration. Blood.

[32] Zhang F., Tsai S., Kato K., Yamanouchi D., Wang C., Rafii S., Liu B., Kent K.C. (2009). Transforming growth factor-β promotes recruitment of bone marrow cells and bone marrow-derived mesenchymal stem cells through stimulation of MCP-1 production in vascular smooth muscle cells. J. Biol. Chem..

[33] Cheng J., Diaz Encarnacion M.M., Warner G.M., Gray C.E., Nath K.A., Grande J.P. (2005). TGF-beta1 stimulates monocyte chemoattractant protein-1 expression in mesangial cells through a phosphodiesterase isoenzyme 4-dependent process. Am. J. Physiol. Cell Physiol..

[34] Gorbacheva A.M., Uvarova A.N., Ustiugova A.S., Bhattacharyya A., Korneev K.V., Kuprash D.V., Mitkin N.A. (2021). EGR1 and RXRA transcription factors link TGF-β pathway and CCL2 expression in triple negative breast cancer cells. Sci. Rep..

[35] Benitez-Garrido J.P., Ibarra-Sanchez A., Macías-Silva M., Villalobos-Molina R., Padilla-Trejo J.A., Gonzalez-Espinoza C. (2009). TGFbeta Presence During IgE-dependent Sensitization Primes Mast Cells for Higher VEGF Production After FcRI Activation. Open Allergy J..

[36] Zhao W., Gomez G., Yu S.-H., Ryan J.J., Schwartz L.B. (2008). TGF-beta 1 Attenuates Mediator Release and De Novo Kit Expression by Human Skin Mast Cells through a Smad-Dependent Pathway. J. Immunol..

[37] Ndaw V.S., Abebayehu D., Spence A.J., Paez P.A., Kolawole E.M., Taruselli M.T., Caslin H.L., Chumanevich A.P., Paranjape A., Baker B. (2017). TGF-β1 Suppresses IL-33–Induced Mast Cell Function. J. Immunol..

[38] Frigeri L., Apgar J.R. (1999). The Role of Actin Microfilaments in the Down-Regulation of the Degranulation Response in RBL-2H3 Mast Cells. J. Immunol..

[39] Ramírez-Valadez K.A., Vázquez-Victorio G., Macías-Silva M., González-Espinosa C. (2017). Fyn kinase mediates cortical actin ring depolymerization required for mast cell migration in response to TGF-β in mice. Eur. J. Immunol..

[40] Edlund S., Landström M., Heldin C.H., Aspenstrom P. (2002). Transforming growth factor-beta-induced mobilization of actin cytoskeleton requires signaling by small GTPases Cdc42 and RhoA. Mol. Biol. Cell.

[41] Soond S.M., Everson B., Riches D.W., Murphy G. (2005). ERK-mediated phosphorylation of Thr735 in TNF-α-converting enzyme and its potential role in TACE protein trafficking. J. Cell Sci..

[42] Rousseau S., Papoutsopoulou M., Symons A., Cook D., Lucocq J.M., Prescott A.R., Cohen P. (2008). TPL2-mediated activation of ERK1 and ERK2 regulates the processing of pre-TNF-α in LPS-stimulated macrophages. J. Cell Sci..

[43] Kato K., Kikuchi S., Shubayev V.I., Myers R.R. (2009). Distribution and tumor necrosis factor-alpha isoform binding specificity of locally administered etanercept into injured and uninjured rat sciatic nerve. Neuroscience.

[44] Grattendick K.J., Nakashima J.M., Feng L., Giri S.N., Margolin S.B. (2008). Effects of three anti-TNF-α drugs: Etanercept, infliximab and pirfenidone on release of TNF-α in medium and TNF-α associated with the cell in vitro. Int. Immunopharmacol..

[45] Falcone F.H., Wan D., Barwary N., Sagi-Eisenberg R. (2018). RBL cells as models for in vitro studies of mast cells and basophils. Immunol. Rev..

[46] Kuburich N.A., Sabapathy T., Demestichas B.R., Maddela J.J., den Hollandera P., Mani S.A. (2023). Proactive and reactive roles of TGF-β in cancer. Semin. Cancer Biol..

[47] Akin C., Siebenhaar F., Wechsler J.B., Youngblood B.A., Maurer M. (2024). Detecting Changes in Mast Cell Numbers Versus Activation in Human Disease: A Roadblock for Current Biomarkers?. J. Allergy Clin. Immunol. Pract..

[48] Olsson N., Piek E., ten Dijke P., Nilsson G. (2000). Human mast cell migration in response to members of the transforming growth factor-beta family. J. Leukoc. Biol..

[49] Gomez G., Ramirez C.D., Rivera J., Patel M., Norozian F., Wright H., Kashyap M.V., Barnstein B.O., Fischer-Stenger K., Schwartz L.B. (2005). TGF-β1 Inhibits Mast Cell FcεRI Expression. J. Immunol..

[50] Fernando J.F., Faber T.W., Pullen N., Falanga Y.T., Kolawole E.M., Oskeritzian C.A., Barnstein B.O., Bandara G., Li G., Schwartz L.B. (2013). Genotype-Dependent Effects of TGF-β1 on Mast Cell Function: Targeting the Stat5 Pathway. J. Immunol..

[51] Yoshimura T. (2018). The chemokine MCP-1 (CCL2) in the host interaction with cancer: A foe or ally?. Cell Mol. Immunol..

[52] Feinberg M.W., Shimizu K., Lebedeva M., Haspel R., Takayama K., Chen Z., Frederick J.P., Wang X.F., Simon D.I., Libby P. (2004). Essential Role for Smad3 in Regulating MCP-1 Expression and Vascular Inflammation. Circ. Res..

[53] Langert K.A., Von Zee C.L., Stubbs E.B. (2013). Cdc42 GTPases facilitate TNF-α-mediated secretion of CCL2 from peripheral nerve microvascular endoneurial endothelial cells. J. Peripher. Nerv. Syst..

[54] Xia L., Lu J., Xiao W. (2011). Blockage of TNF-α by infliximab reduces CCL2 and CCR2 levels in patients with rheumatoid arthritis. J. Investig. Med..

[55] Medina-Tamayo J., Ibarra-Sánchez A., Padilla-Trejo A., Gonzalez-Espinosa C. (2011). IgE-dependent sensitization increases responsiveness to LPS but does not modify development of endotoxin tolerance in mast cells. Inflamm. Res..

[56] Sandig H., Bulfone-Paus S. (2012). TLR signaling in mast cells: Common and unique features. Front. Immunol..

[57] Madera-Salcedo I.K., Cruz S.L., Gonzalez-Espinosa C. (2013). Morphine prevents lipopolysaccharide-induced TNF secretion in mast cells blocking IκB kinase activation and SNAP-23 phosphorylation: Correlation with the formation of a β-arrestin/TRAF6 complex. J. Immunol..

[58] Guzman-Mejia F., López-Rubalcava C., González-Espinoza C. (2018). Stimulation of nAchRα7 Receptor Inhibits TNF Synthesis and Secretion in Response to LPS Treatment of Mast Cells by Targeting ERK1/2 and TACE Activation. J. Neuroimmune Pharmacol..

[59] Hamby M.E., Gragnolati A.R., Hewett S.J., Hewett J.A. (2008). TGFβ1 and TNFα potentiate nitric oxide production in astrocyte cultures by recruiting distinct subpopulations of cells to express NOS-2. Neurochem. Int..

[60] McGee D.W., Bamberg T., Vitkus S.J., McGhee J.R. (1995). A synergistic relationship between TNF-alpha, IL-1 beta, and TGF-beta 1 on IL-6 secretion by the IEC-6 intestinal epithelial cell line. Immunology.

[61] Wang Z., Franke K., Zuberbier T., Babina M. (2022). Cytokine stimulation by MRGPRX2 occurs with lower potency than by FceRI aggregation but with similar dependence on the extracellular signal-regulated kinase 1/2 module in human skin mast cells. J. Investig. Dermatol..

[62] Katanov C., Lerrer S., Liubomirski Y., Leider-Trejo L., Meshel T., Bar J., Feniger-Barish R., Kamer I., Soria-Artzi G., Kahani H. (2015). Regulation of the inflammatory profile of stromal cells in human breast cancer: Prominent roles for TNF-a and the NFkB pathway. Stem Cell Res. Ther..

[63] Yip A.J.W., Lee Y.Z., Kow A.S.F., Wong C.S.A., Lee M.T., Tham C.L., Tan J.W. (2025). Current utilization trend of immortalized mast cell lines in allergy research: A systematic review. Immunol. Res..

[64] Ayo T.E., Adhikari P., Xu H. (2023). TNFR1 links TNF exocytosis to TNF production in allergen-activated RBL-2H3 cells. Cell Signal.

[65] Zunke F., Rose-John S. (2017). The shedding protease ADAM17: Physiology and pathophysiology. Biochim. Biophys. Acta Mol. Cell Res..

[66] de Queiroz T.M., Lakkappa N., Lazartigues E. (2020). ADAM17-Mediated Shedding of Inflammatory Cytokines in Hypertension. Front. Pharmacol..

[67] Pahima H.T., Dwyer D.F. (2025). Update on mast cell biology. J. Allergy Clin. Immunol..

[68] Kannen V., Grant D.M., Matthews J. (2024). The mast cell-T lymphocyte axis impacts cancer: Friend or foe?. Cancer Lett..

[69] Savage A., Risquez C., Gomi K., Schreiner R., Borczuk A.C., Worgall S., Silver R.B. (2023). The mast cell exosome-fibroblast connection: A novel pro-fibrotic pathway. Front. Med..

[70] Meurer S.K., Neß M., Weiskirchen S., Kim P., Tag C.G., Kauffmann M., Huber M., Weiskirchen R. (2016). Isolation of mature (Peritoneum-Derived) mast cells and immature (Bone Marrow- Derived) mast cell precursors from mice. PLoS ONE.

[71] Qiao Y., Chen J., Lim Y.B., Finch-Edmondson M.L., Seshachalam V.P., Qin L., Jiang T., Low B.C., Singh H., Lim C.T. (2017). YAP Regulates Actin Dynamics through ARHGAP29 and Promotes Metastasis. Cell Rep..

[72] Wakasuki T., Schwab B., Thompson N.C., Elson E.L. (2001). Effects of cytochalasin D and latrunculin B on mechanical properties of cells. J. Cell Sci..

