# Supplementary material for: An Autocrine Regulator Loop Involving Tumor Necrosis Factor and Chemokine (C-C motif) Ligand-2 Is Activated by Transforming Growth Factor-β in Rat Basophilic Leukemia-2H3 Mast Cells
Source: Int J Mol Sci. 2025 Apr 30;26(9):4263. doi: 10.3390/ijms26094263 (PMC12071771; doi:10.3390/ijms26094263)

**Supplementary Figure S1.** TGF-β did not cause degranulation in BMMCs or RBL-2H3 mast cells. RBL-2H3 and BMMCs were stimulated for 1 h with 300 ng/ml IgE/Ag (IgE + DNP), 100 pM TGF-β, 1 mM LatB or TGF-β plus LatB, and β-hexosaminidase release was measured as described in supplementary method.

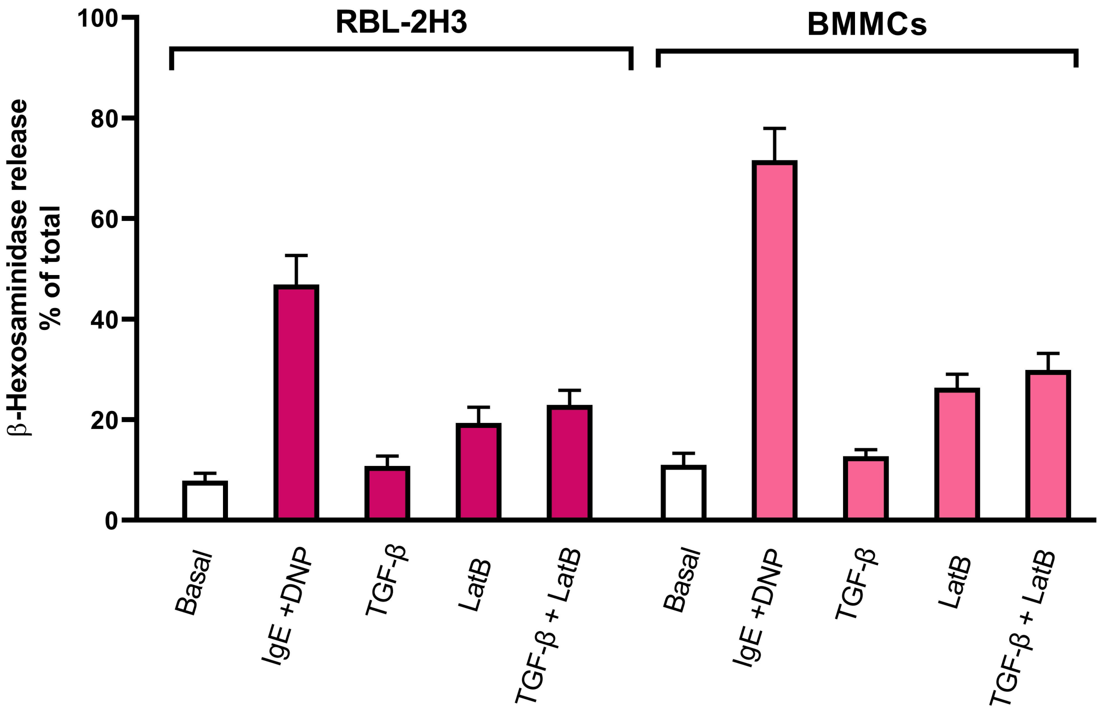


**Supplementary method for Figure S1:** *Degranulation assay*

Degranulation was measured determining the amount of **β**-hexosaminidase activity in the supernatants of cells, as described previously [Benitez-Garrido et al. 2009]. Light absorbance was measured at 405 nm in an ELISA plate reader. The amount of **β**-hexosaminidase release was determined by dividing the activity observed in the supernatant by the total activity of the enzyme, detected in the supernatant plus cell pellet [Benitez-Garrido et al. 2009].

**Supplementary Figure S2.** An antagonist of CCR2 did not affect the TGF-β-induced secretion of pre-formed CCL-2 chemokine in RBL-2H3 mast cells. RBL-2H3 cells were pre-treated for 30 min without or with 10 μM BMS CCR2 22 (antagonist of CCR2 receptor), and then treated for 1 h with 100 pM TGF-β. CCL-2 concentration was measured in conditioned media by ELISA.

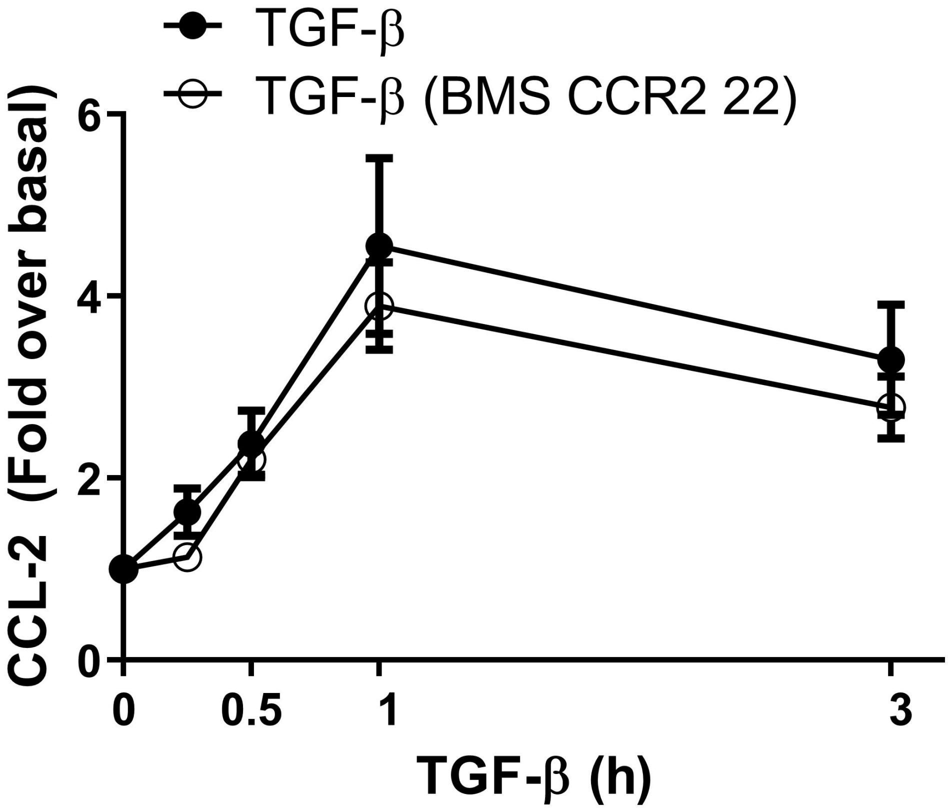

**Supplementary Figure S3:** TGF-β-induced CCL-2 secretion partially requires the activation of an autocrine TNF signaling in RBL-2H3. RBL-2H3 cells were pre-treated for 30 min with 50 μM or 100 μM UCB-9260 (TNFR inhibitor), and then treated for 1 h with 100 pM TGF-β. CCL-2 concentration was measured in conditioned media by ELISA. Graphs show the mean ± SEM (n=4).

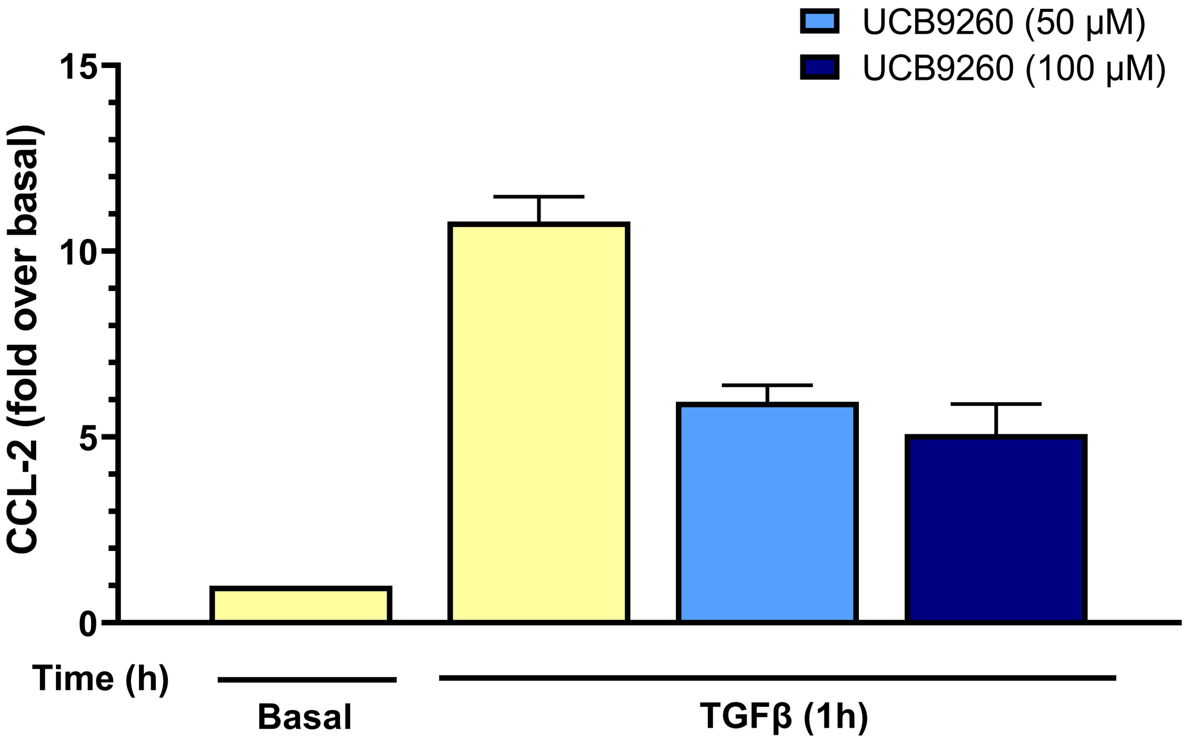

**Supplementary Figure S4.** Long-treatment of RBL-2H3 mast cells with midostaurin to inhibit KIT receptor blocked TGF-β-induced TNF secretion. RBL-2H3 cells were pre-treated with 10 μM midostaurin (Mid) at different times, and then treated for 1 h with 100 pM TGF-β. TNF concentration was measured in conditioned media by ELISA. Graphs show the mean ± SEM (n=4).

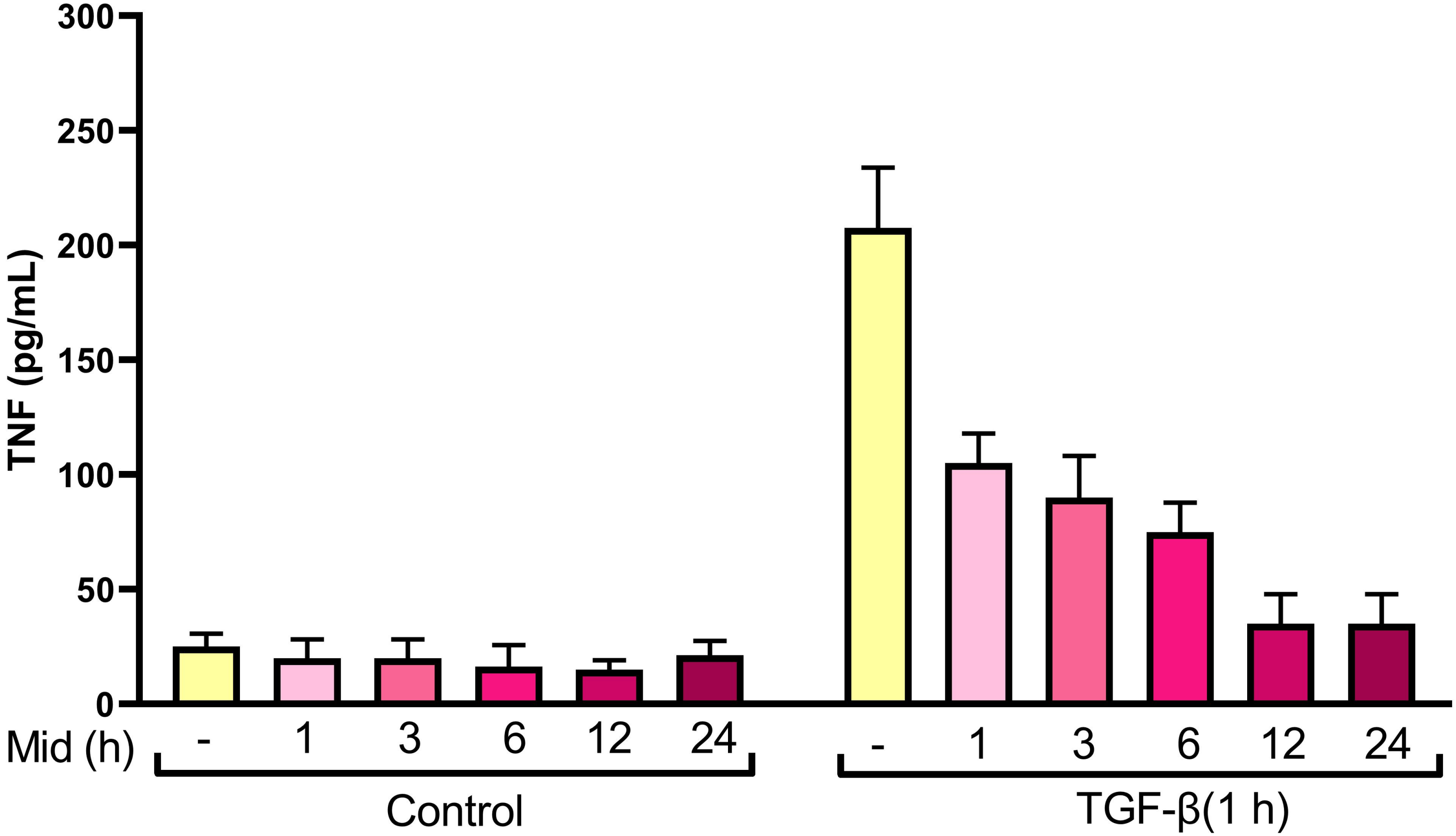

Supplement: Supplementary file 1 [file ijms-26-04263-s001.zip › ijms-3569042-supplementary.pdf]
